# Continued overexpression of *EPSPS* transgene enhances fitness in multigeneration crop–wild rice hybrids and its long-term environmental impact

**DOI:** 10.3389/fpls.2026.1707859

**Published:** 2026-03-09

**Authors:** Xiao-Qi Jiang, Yang Cao, Xu-Peng Wang, Li-Li Chang, Hua Kong, Yu-Liang Zhang, Xia Zhou, Bao-Rong Lu

**Affiliations:** 1 Institute of Tropical Bioscience and Biotechnology, Sanya Research Institute of Chinese Academy of Tropical Agricultural Sciences, Sanya, China; 2 State Key Laboratory of Wetland Conservation and Restoration, National Observations and Research Station for Wetland Ecosystems of the Yangtze Estuary, School of Life Sciences, Fudan University, Shanghai, China

**Keywords:** advanced hybrid generation, environmental biosafety, fitness benefit, *Oryza rufipogon*, risk assessment, transgene flow

## Abstract

Transgene flow from genetically engineered (GE) crops to their wild relatives has aroused worldwide biosafety concerns. Environmental impact caused by transgene flow from GE glyphosate herbicide-tolerant rice overexpressing the endogenous *EPSPS* (5-enolpyruvylshikimate-3-phosphate synthase) transgene to its wild relatives has become a great concern. This is due to the significantly increased fitness conferred by the transgene in crop–wild hybrids. Whether the increased fitness is maintained consistently in the advanced generations of the hybrid progenies is unclear and needs science-based evaluation. Such evaluation plays an essential role in understanding the potential environmental impact caused, particularly by the *EPSPS* transgene. We therefore examined the relative expression quantities (REQs) of the *EPSPS* (trans)gene and fitness-related traits in advanced F_3_–F_5_ hybrid lineages derived from crosses between GE *EPSPS* rice (*Oryza sativa*) and two wild rice (*Oryza rufipogon*) populations in common garden experiments. Our results showed that the crop–wild rice hybrid lineages containing the transgene have significantly higher REQs of the *EPSPS* (trans)gene and increased values of the fitness-related traits, including the number of tillers, panicles, and seeds per plant. In addition, the fitness benefit, conferred by overexpressing the *EPSPS* transgene, presented consistently in GE F_3_–F_5_ hybrid lineages. Consequently, the overexpression of the rice endogenous *EPSPS* transgene may consistently increase the fitness of crop–wild rice hybrid progenies in advanced generations, which may promote the persistence and spread of the transgene in wild rice populations. Thus, assessing the long-term environmental impact caused by the outflow of the glyphosate-tolerant *EPSPS* transgene from GE rice should be seriously considered.

## 1 Introduction

Over the last decades, more than 3.4 billion hectares of genetically engineered (GE) crops containing herbicide resistance, insect resistance, and other types of transgenes have been cultivated around the world, which contributed significantly to the global food security (Cheng et al., 2024). However, the extensive environmental releases and commercial applications of GE crops have aroused great concerns over biosafety issues worldwide, including the potential food safety and environmental impact (Hall et al., 2000; Stewart et al., 2000; Kuiper et al., 2001; Wang and Lu, 2025). A transgene that moves from GE crops to the populations of crop wild relative species through pollen-mediated gene flow may persist and spread in the wild populations (Lu and Snow, 2005; Lu, 2008). Such a process of transgene incorporating into and spreading in the populations of wild relative species may cause unwanted environmental consequences or impacts, which represents one of the most concerning environmental biosafety issues (Lu, 2008; Lu et al., 2016; Pandolfo et al., 2018; Vazquez-Barrios et al., 2021; Caradus, 2023).

Many studies have provided a large amount of evidence on the relatively high frequency of gene flow from some crop species to their wild relative populations, suggesting that there is a great possibility of pollen-mediated transgene flow (Kim et al., 2019; Song et al., 2003; Sun et al., 2015; Wang et al., 2014; Zhang et al., 2018; Liu et al., 2020; Jhala et al., 2021). Consequently, the likelihood of environmental problems caused by the transgene flow from GE crops to their wild relative species will be relatively high. Based on these observations, many more studies have been conducted to determine the potential consequences or impacts of pollen-mediated transgene flow from different crop species to their wild or weedy populations. These studies have included the estimation of transgene expression in crop–wild populations (Xia et al., 2009; Wang et al., 2014; Yang et al., 2017a; Fang et al., 2018; Lohn et al., 2021) and its resulting fitness effect (benefit and cost) in crop–wild hybrid populations containing a transgene (Snow et al., 2003; Londo et al., 2010; Liu et al., 2016; Wang et al., 2014; Sagers et al., 2015; Li et al., 2016; Yang et al., 2015, 2017b; Magomere et al., 2016).

It is generally recognized that the extensive cultivations of GE crops containing herbicide-resistant transgenes may create highly tolerant weeds if such herbicide-resistant transgene move to and incorporated into wild or weedy relative populations through natural hybridization (Brimner et al., 2005; Lu and Snow, 2005; Beckie et al., 2006; Lu, 2008; Lu and Yang, 2009; Londo et al., 2010). This is because the herbicide-resistant transgenes may bring considerable fitness benefit to the wild or weedy populations under the environmental conditions with herbicide-selection pressure (Harker et al., 2006; Londo et al., 2010; Sagers et al., 2015; Yang et al., 2015; Zhang et al., 2023). In fact, some studies have further indicated that different transgenes, particularly those with herbicide resistance, can significantly increase the fitness of the crop–wild/weed hybrids with or even without the selection pressure (Liu et al., 2014; Wang et al., 2014; Yang et al., 2017b; Yook et al., 2021). Therefore, biosafety or risk assessments of these transgenes should be seriously considered to understand their potential environmental impacts in agroecosystems with a strong selection pressure and the natural ecosystems where selection pressure may be free or not strong enough.

Usually, comparative studies of the fitness effect (benefit or cost) caused by a transgene have focused on the early generations (e.g., F_1_, F_2_, or BC_1_) hybrid lineages produced from the artificial crosses between a GE crop and wild relative species in common garden experiments (Burke and Rieseberg, 2003; Wang et al., 2014; Sagers et al., 2015; Magomere et al., 2016; Yang et al., 2017b). However, only very few studies have reported the fitness effect of the transgenes in the advanced generations (F_3_ and higher) of hybrid lineages (Dong et al., 2011; Yang et al., 2015). Under the assumption that the expression and fitness effect of an incorporated gene may gradually shift with genetic recombination and decrease in heterosis in advanced-generation hybrid progenies (Rieseberg et al., 1999, 2003; Lippman and Zamir, 2007), the estimation of the consistency and stability of the expression and fitness effect in subsequent hybrid generations after F_2_ has become essential for the long-term environmental impact of transgene flow (Snow et al., 2010). Only if the expression and fitness effect of a transgene do not decline in the advanced-generation hybrid progenies will transgene flow from GE crops to their wild relative populations then cause long-term environmental impacts (see the explanation in **Supplementary Figure 1**).

As an important staple food that is cultivated globally and consumed by more than half of the world’s population, the research and development of genetic engineering of rice (*Oryza sativa*) are highly active, with great progress (Achary et al., 2020; Javed et al., 2024). Likewise, studies associated with GE rice biosafety, including environmental impacts, have been undertaken (Lu and Snow, 2005; Lu and Yang, 2009; Li et al., 2024; Xie et al., 2024). Previous studies have been conducted to estimate the potential environmental impact of transgene flow from glyphosate herbicide-tolerant GE rice overexpressing the rice endogenous *EPSPS* (5-enolpyruvylshikimate-3-phosphate synthase) transgene (Su et al., 2008; Achary et al., 2020; Wang et al., 2014; Yang et al., 2017b). In addition to the increased glyphosate tolerance, results from some of these studies unexpectedly suggested that the overexpression of the *EPSPS* transgene significantly increased the biosynthesis of tryptophan, phenylalanine, tyrosine, chlorophyll, and lignin in the transgenic F_1_ and F_2_ hybrid lineages derived from crosses between *EPSPS* GE rice and weedy rice (*O. sativa* f. *spontanea*) or wild rice (*Oryza rufipogon*) (Wang et al., 2014; Ma et al., 2020; Wu et al., 2020). Consequently, the fecundity-related traits, such as the number of tillers, panicles, and seeds per plant, were significantly increased in these F_1_ and F_2_ hybrid lineages containing the transgene in the herbicide-free environment (Wang et al., 2014; Yang et al., 2017b). In addition, similar results were also obtained, showing considerable increases in auxin, biomass, and other fecundity-associated traits in the GE *Arabidopsis* lineages containing the rice *EPSPS* transgene under the herbicide-free condition (Fang et al., 2018). However, little is known about the stability of *EPSPS* transgene overexpression and the fitness benefit in advanced generations of crop–weedy/wild hybrid progenies. Such information is critical for the biosafety assessment of the long-term environmental impact caused by *EPSPS* transgene flow to weedy and wild relative populations.

To understand the potential long-term fitness benefit conveyed by overexpressing the *EPSPS* transgene in advanced generations of hybrid progenies, we produced F_3_–F_5_ crop–wild hybrid lineages derived from an *EPSPS* transgenic rice line crossed with two wild rice populations (Yang et al., 2017b). We examined the expression of the *EPSPS* transgene and the fitness-related traits of the GE and non-GE hybrid lineages in the common garden experiments. We also analyzed the correlation between the *EPSPS* gene expression and fitness-related traits of the GE and non-GE hybrid lineages. The primary objectives of this study were to address the following questions. i) Does the *EPSPS* transgene overexpress consistently in advanced generations of the GE crop–wild rice hybrid progenies? ii) Does the *EPSPS* transgene provide consistent fitness benefits to the GE crop–wild rice hybrid progenies in advanced generations? iii) Is the overexpression of the *EPSPS* (trans)gene correlated with the enhanced fitness benefit in the GE hybrid progenies? The answers to these questions can help us to estimate whether the *EPSPS* transgene can persist and spread in wild rice populations, resulting in ecological and environmental impacts in the case of *EPSPS* transgene flow.

## 2 Materials and methods

### 2.1 Plant materials

Homozygous transgenic (GE) hybrid lineages, their non-transgenic (non-GE) counterparts, the parental GE rice line (EP3), a non-GE rice variety (MH86), and two common wild rice populations (w1 and w2) were included in this study. The homozygous GE and non-GE crop–wild rice hybrid lineages were derived from crosses between EP3 (as pollen donor) and wild rice populations (as pollen recipient) (see **Supplementary Figure 2**). The identification of the presence or absence of the *EPSPS* transgene in hybrid plants was performed using the *EPSPS* transgene as a molecular fingerprint, following the method of Yang et al. (2017b). Approximately 20 plants each from the two wild rice populations were included to produce the crop–wild rice hybrids (Yang et al., 2017b). Approximately 100 F_1_ plants from each hybrid combination were randomly selected to form a bulk population for developing different generations of hybrid progenies through successive self-pollination. The GE and non-GE homozygotes on the transgene locus were identified and selected by detecting the genotype of 15 offspring plants; i.e., the offspring of a homozygote did not segregate on the locus. Only the F_3_–F_5_ hybrid progenies, which were bred and identified in our study based on the F_2_ materials of Yang et al. (2017b), were included in further experiments. The EP3 line of the GE rice parent was developed from the non-GE rice variety MH86, of which the endogenous *EPSPS* gene was engineered to overexpress EPSPS to tolerate the glyphosate herbicide (Su et al., 2008). The two wild rice populations were collected from Dongxiang (w1) in Jiangxi Province and Suixi (w2) in Guangdong Province, China.

### 2.2 Determination of *EPSPS* gene and its expression

The real-time PCR of cDNA was conducted to determine the relative expression quantities (REQs) of the endogenous *EPSPS* gene in non-GE crop–wild hybrid and MH86 plants, in addition to the determination of the endogenous *EPSPS* gene and transgene in the GE crop–wild hybrid and GE EP3 plants. For RNA extraction, fresh leaf tissues from six mixed-leaf samples (replicates) each containing the leaf tissues from six plants grown in a greenhouse were collected at two growth stages after seeds were germinated for 60 and 90 days (defined as 60- or 90-day growth stage). Sampling was conducted at two critical developmental stages, which are key determinants of fitness throughout the life cycle of rice plants. Variation in *EPSPS* gene expression at these stages may reflect overall physiological differences between GE and non-GE control plants. All sampled plants were cultivated in a greenhouse, separate from the common garden field experiment. The methods of RNA extraction followed the description of the RNAprep Pure Plant Kit [TianGen Biotech (Beijing) Co., Ltd., Beijing, China]. The reverse transcription from RNA to cDNA solution was made using the PrimeScript RT Reagent Kit (TaKaRa Biotech Co., Ltd., Dalian, China).

In the real-time PCR experiment, the *EPSPS* gene-specific primer pair (forward: GCAGTTGGACCATCAGCGAAG; reverse: CTGTTGAGAAGGATGCGAAAGA) designed by Wang et al. (2014) was used to amplify the transcripts (relative expression quantities) for both the endogenous *EPSPS* gene and the transgene in the plant materials. In addition, the actin gene-specific primer pair (forward: TCCATCTTGGCATCTCTCAG; reverse: GTACCCGCATCAGGCATCTG) was used to amplify the transcripts (expression quantities) of the actin gene that was used as the reference gene. The expression quantities of the endogenous *EPSPS* gene and the transgene were adjusted using the absolute expression quantities of the *EPSPS* (trans)gene and the actin gene. The two sets of primer pairs were synthesized by the Shanghai Personal Gene Technology Co., Ltd. (Shanghai, China).

For the real-time PCR procedure, 1 μL of template cDNA was mixed with 0.4 μL each of the forward and reverse primers (10 mmol/L) and 10 μL of 2× SYBR real-time PCR premixture (Vazyme Biotech Co., Ltd., Nanjing, China) in a final volume of 20 μL. The PCR was conducted at 15 s at 95°C and 30 s at 60°C for 40 cycles using the TIB8600 real-time fluorescence quantitative PCR instrument (Triplex International Biosciences Co., Ltd., Xiamen, China). The expression quantity of one sample of non-GE cultivated rice MH86 was randomly selected and set as 1 (control) to calculate the relative expression quantities of other samples.

### 2.3 Design of the common garden field experiment

The common garden field experiment was conducted to estimate the fitness effect caused by the *EPSPS* transgene in wild rice populations, supposedly through gene flow, by the comparison between the GE and non-GE crop–wild rice hybrid lineages, in addition to wild rice parents that were also used as a control. Cultivated parental lines EP3 and MH86 were not included in the common garden field experiment. Seedlings of the GE, non-GE hybrid lineages (F_3_–F_5_), and wild rice parents were developed in a growth chamber (27°C, 16-h light and 8-h dark) under the liquid culture condition (Yoshida et al., 1972). Seedlings at the ~30-day growth stage were transplanted into a rice field with two cultivation designs: the pure-planting plots (broad spacing and less competition between plants) and mixed-planting plots (narrow spacing and stronger competition between plants).

In the pure-planting plots, 36 randomly selected plants from either the GE or non-GE hybrid lineage were planted in a 6 × 6 grid with 50-cm spacing (low competition). In the mixed-planting plots, 36 randomly selected plants, 18 each from GE hybrid lineages and their corresponding non-GE counterparts, were planted alternatingly in a 6 × 6 grid with 30-cm spacing (strong competition). In addition, 36 randomly selected plants from the two wild parental populations (w1 and w2) were also planted in plots with a 6 × 6 grid and 50-cm spacing, following the pure-planting design. Six plots (replicates) were included for each treatment, including two cultivation designs and 10 materials. Consequently, a total of 120 plots with 4,320 plants were arranged in the field experiment with a completely randomized field layout. The experiment was conducted in a designated Biosafety Assessment Center of Fujian Academy of Agricultural Sciences in Fuzhou, Fujian Province, China.

Five fitness-related traits were measured, including plant height, the number of tillers, the number of panicles, the number of filled seeds per plant, 1000-seed weight, and over-winter plant survival ratios (**Table 1**) in the common garden field experiment. The trait of the over-winter plant survival ratios was only measured in the pure-planting plots. Only 25 plants in the middle rows of the pure-planting plots were measured for the fitness-related traits, whereas all 18 GE and non-GE plants were measured in the mixed-planting plots.

**Table 1:** The fitness-related traits measured in the common garden field experiment and their method of measurement.

Trait	Method of measurement
Plant height (cm)	Height from ground to the flag leaf tip of main tiller measured at 60, 90, and 120 days
Number of tillers per plant	Tiller number of a plant measured at 60, 90, and 120 days
Number of panicles per plant	Panicle number of a plant measured at maturity
Number of filled seeds per plant	Number of filled seeds of a plant measured after maturity
1000-seed weight (g)	Weight of 1,000 randomly sampled seeds from the same plant
Over-winter plant survival ratios (%)	Ratio of the plants that survived through winter

### 2.4 Statistical analysis

Three-way ANOVAs (Stewart, 1973) were performed on GE and non-GE F_3_–F_5_ hybrid progenies with w1 or w2 parents to determine the effect of the transgene (T), generation (G), and hybrid combination with different wild parents (WP) on the REQs of *EPSPS* and the performances of the fitness-related traits. The independent-samples Student’s *t*-tests (Blalock, 1972) were conducted to test the differences in REQs of the *EPSPS* (trans)gene between GE and non-GE hybrid rice lineages and between GE rice (EP3) and non-GE rice (MH86) lines, and fitness-related traits between GE and non-GE rice lineages in the pure-planting plots, in addition to the mean performances of the traits between the GE hybrid lineages of different generations (F_3_, F_4_, and F_5_) and their parental wild rice plants in the pure-planting plots. The paired-samples Student’s *t*-tests (Blalock, 1972) were conducted to test the differences in fitness-related traits between GE and non-GE rice lineages in the mixed-planting plots.

In addition, Pearson’s tests and linear regressions (Blalock, 1972) were conducted to analyze the correlation between the REQs of the *EPSPS* (trans)gene and the relative performance of the fitness-related traits in all F_3_–F_5_ crop–wild rice hybrid lineages. The relative performance was determined as the ratios between the fitness-related traits of different hybrid lineages and the traits of the best-performing hybrid lineage in this study.

## 3 Results

### 3.1 Relative expression quantities of the endogenous *EPSPS* gene and transgene

The three-way ANOVAs were performed to determine the impact of the key three factors: transgene (T), generation (G), and hybrid combination with different wild parents (WP) on the REQs of the *EPSPS* (trans)genes. Results showed a significant effect (*p* < 0.001) of the *EPSPS* transgene on REQs in the F_3_–F_5_ crop–wild rice hybrid lineages at the 60- and 90-day growth stages (**Table 2**). However, the results did not show a significant effect (*p* > 0.05) of generations (F_3_*vs*. F_4_*vs*. F_5_), wild rice parents (w1 *vs*. w2), and the interaction of the three factors on REQs in the obtained hybrid lineages (**Table 2**). Altogether, the results demonstrated the significant role of the *EPSPS* transgene in maintaining a high level of *EPSPS* expression in the F_3_–F_5_ crop–wild hybrid lineages, although there were no significant influences of the other two factors (G and WP) on *EPSPS* expression.

**Table 2:** Three-way ANOVAs for the effects of transgene (transgenic *vs*. non-transgenic), generations (F_3_*vs*. F_4_*vs*. F_5_), and wild rice parents (w1 *vs*. w2) on the relative expression quantities (REQs) of *EPSPS* at the 60- and 90-day growth stages in F_3_–F_5_ crop–wild rice hybrid lineages.

Factors	df^1^	60-day		90-day	
		*F* ^2^	*p* ^3^	*F*	*p*
Transgene (T)	1	331.6	**<0.001** ^4^	640.0	**<0.001**
Generation (G)	2	0.8	0.462	1.9	0.157
Wild parent (WP)	1	0.3	0.589	1.4	0.236
T × G	2	0.7	0.511	1.8	0.167
T × WP	1	0.3	0.584	1.5	0.224
G × WP	2	1.1	0.338	1.8	0.169
T × G × WP	2	1.0	0.357	1.9	0.166

^1^df indicates degree of freedom.

^2^*F* indicates *F* value.

^3^*p* indicates level of significance.

^4^Bold letters indicate significant differences at the level of 0.05.

The independent-samples Student’s *t*-tests were performed to determine the differences caused by the transgene on *EPSPS* REQs between the GE and non-GE crop–wild rice hybrid lineages. Results from the independent-samples *t*-test showed significantly higher levels (*p* < 0.01) of *EPSPS* REQs in the GE hybrid lineages than in the non-GE lineages at both the 60- and 90-day growth stages (**Table 3**). Similarly, the independent-samples *t*-tests also indicated a significantly higher level (*p* < 0.05) of *EPSPS* REQs in the GE cultivated rice line (EP3) than in its non-GE rice counterpart (MH86) at the same growth stages, suggesting the effectiveness of the transgene on *EPSPS* REQs. These results indicated that the *EPSPS* transgene derived from the GE cultivated rice line (EP3) promoted *EPSPS* expression in the crop–wild rice hybrid lineages in different generations (F_3_, F_4_, and F_5_).

**Table 3:** Comparison of the relative expression quantities (REQs) of *EPSPS* between the engineered (GE) and non-GE cultivated rice (GE EP3 *vs*. non-GE MH86) and F_3_–F_5_ crop–wild rice hybrid lineages based on the independent-samples Student’s *t*-test.

Plant lines	60 days		90 days	
	GE	Non-GE	GE	Non-GE
Cultivated rice				
EP3/MH86	80.0 ± 14.0^1^	1.4 ± 0.2 ^**2^	72.0 ± 19.5	1.2 ± 0.1 ^*^
Hybrid lineage				
w1-F_3_	67.0 ± 11.9	1.3 ± 0.6 ^***^	151.5 ± 15.6	1.6 ± 0.7 ^***^
w1-F_4_	82.7 ± 9.4	1.1 ± 0.5 ^***^	174.6 ± 16.6	1.4 ± 0.5 ^***^
w1-F_5_	94.9 ± 10.6	2.0 ± 0.6 ^***^	199.8 ± 18.0	1.1 ± 0.6 ^***^
w2-F_3_	78.1 ± 11.8	1.3 ± 0.4 ^***^	178.0 ± 6.5	0.7 ± 0.2 ^***^
w2-F_4_	77.2 ± 7.8	1.7 ± 0.4 ^***^	220.7 ± 17.5	1.6 ± 0.6 ^***^
w2-F_5_	75.4 ± 10.5	1.6 ± 0.6 ^***^	179.8 ± 26.2	1.2 ± 0.4 ^**^

^1^Numbers followed by the averages indicate the standard error (n = 6 replicates).

^2^Comparisons were made between the genetically engineered (GE) and non-GE hybrid lineages based on the independent-samples Student’s *t*-test.

^*^p < 0.05; ^**^p < 0.01; ^***^p < 0.001.

### 3.2 Increased fitness benefits in GE F_3_–F_5_ crop–wild rice hybrid lineages overexpressing *EPSPS*

Three-way ANOVAs were performed to determine the impact of the transgene (T), generations (G), and wild rice parents (WP) on the fitness-related traits, including plant height, the number of tillers, the number of panicles, the number of filled seeds per plant, 1000-seed weight, and the over-winter plant survival ratios at different plant growth stages. Results showed the significant effect (*p* < 0.01) of the *EPSPS* transgene only on these fitness-related traits, including the number of tillers at the 120-day stage and the number of panicles and filled seeds per plant in the pure-planting plots (**Table 4**). In addition, results also showed the significant effect (*p* < 0.01) on more traits, including the plant height at the 120-day stage, the number of tillers at the 90- and 120-day stages, and the number of panicles and filled seeds per plant in the mixed-planting plots (**Table 4**). Altogether, these results suggested that the *EPSPS* transgene significantly influenced the fitness-related traits of GE crop–wild rice hybrid lineages in the F_3_–F_5_ generations.

**Table 4:** Three-way ANOVAs for the effects of transgene (transgenic *vs*. non-transgenic), generation (F_3_*vs*. F_4_*vs*. F_5_), and wild parent (w1 *vs*. w2) on fitness-related traits of F_3_–F_5_ crop–wild rice hybrid lineages.

Factors	df^1^	Plant height at 60 days (cm)		Plant height at 90 days (cm)		Plant height at 120 days (cm)		No. of tillers per plant at 60 days		No. of tillers per plant at 90 days		No. of tillers per plant at 120 days		No. of panicles per plant		No. of filled seeds per plant		1000-seed weight (g)		Over-winter plant survival ratios (%)	
		*F* ^2^	*p* ^3^	*F*	*p*	*F*	*p*	*F*	*p*	*F*	*p*	*F*	*p*	*F*	*p*	*F*	*p*	*F*	*p*	*F*	*p*
Pure planting																					
Transgene (T)	1	0.8	0.373	1.2	0.285	1.3	0.253	0.4	0.535	0.8	0.374	13.1	**0.001**	63.2	**<0.001**	73.6	**<0.001**	<0.1	0.952	1.9	0.176
Generation (G)	2	5.6	**0.006** ^4^	58.5	**<0.001**	45.7	**<0.001**	5.7	**0.006**	5.0	**0.010**	7.0	**0.002**	1.7	0.193	2.5	0.091	3.6	**0.033**	3.2	**0.049**
Wild parent (WP)	1	1.0	0.326	3.6	0.063	1.8	0.181	1.2	0.282	0.9	0.357	4.2	0.046	0.4	0.507	1.0	**0.002**	6.2	**0.016**	0.8	0.381
T × G	2	1.6	0.208	1.8	0.180	3.2	**0.049**	6.2	**0.003**	<0.1	0.988	0.2	0.859	0.2	0.847	0.1	0.913	0.9	0.425	0.1	0.910
T × WP	1	14.5	**<0.001**	<0.1	0.968	0.1	0.744	6.9	**0.011**	0.1	0.706	0.1	0.719	<0.1	0.917	<0.1	0.942	1.5	0.219	0.1	0.806
G × WP	2	5.6	**0.006**	11.7	**<0.001**	3.7	**0.032**	3.0	**0.057**	5.4	**0.007**	1.8	0.172	3.6	**0.034**	1.6	0.206	2.0	0.144	2.5	0.071
T × G × WP	2	1.6	0.205	5.2	**0.008**	2.6	0.086	0.1	0.915	<0.1	0.979	0.4	0.702	0.4	0.681	0.8	0.473	4.9	**0.011**	1.5	0.231
Mixed planting																					
Transgene (T)	1	<0.1	0.872	2.3	0.132	11.4	**0.001**	0.3	0.607	70.1	**<0.001**	93.8	**<0.001**	52.3	**<0.001**	43.0	**<0.001**	1.3	0.253	-^5^	–
Generation (G)	2	7.7	**0.001**	23.1	<0.001	20.5	**<0.001**	6.7	**0.002**	5.0	**0.010**	4.8	**0.012**	0.1	0.925	9.7	**<0.001**	16.5	**<0.001**	–	–
Wild parent (WP)	1	5.5	**0.022**	5.6	**0.021**	8.3	**0.005**	0.1	0.713	2.6	0.115	8.1	**0.006**	8.1	**0.006**	1.4	0.246	5.7	**0.020**	–	–
T × G	2	1.8	0.169	3.9	**0.027**	2.3	0.106	3.3	0.044	0.8	0.438	1.1	0.346	0.9	0.424	0.7	0.488	0.2	0.826	–	–
T × WP	1	<0.1	0.931	1.9	0.169	0.1	0.799	1.0	0.329	0.7	0.400	1.6	0.208	<0.1	0.961	1.4	0.241	0.7	0.397	–	–
G × WP	2	2.5	0.090	1.3	0.291	2.0	0.144	1.3	0.268	2.3	0.108	0.6	0.541	0.2	0.850	<0.1	0.976	6.9	**0.002**	–	–
T × G × WP	2	0.4	0.687	0.1	0.909	0.8	0.442	3.6	**0.034**	0.1	0.911	0.1	0.911	0.1	0.913	<0.1	0.959	0.1	0.940	–	–

^1^df indicates degree of freedom.

^2^*F* indicates *F* value.

^3^*p* indicates level of significance.

^4^Bold letters indicate significant differences at the level of 0.05.

^5^The trait of over-winter plant survival ratios was not measured in the mixed-planting plots.

The results of the three-way ANOVAs also showed that the factor of generation (F_3_*vs*. F_4_*vs*. F_5_) had a significant effect (*p* < 0.05) on plant height, the number of tillers per plant, 1000-seed weight, and the over-winter plant survival ratios in the pure-planting plots, in addition to plant height, the number of tillers and filled seeds per plant, and 1000-seed weight in the mixed-planting plots (**Table 4**). Our results also showed that the factor of wild rice parents (w1 *vs*. w2) had a significant effect (*p* < 0.05) on the number of filled seeds per plant and 1000-seed weight in the pure-planting plots, in addition to plant height, the number of tillers and panicles per plant, and 1000-seed weight in the mixed-planting plots (**Table 4**). Furthermore, no significant effect (*p* > 0.05) of interaction between the transgene and generations (T × G) was detected on traits on which the transgene had a significant effect, regardless of pure- or mixed-planting models (**Table 4**). Altogether, these results suggested that the considerable fitness effect (benefit) on the traits, such as the number of tillers, panicles, and filled seeds per plant, was more likely driven by continued overexpression of the *EPSPS* transgene in different generations (F_3_, F_4_, and F_5_) of crop–wild hybrid lineages, although some fitness-related traits showed great variation among generations and wild parents.

**Figure 1:**
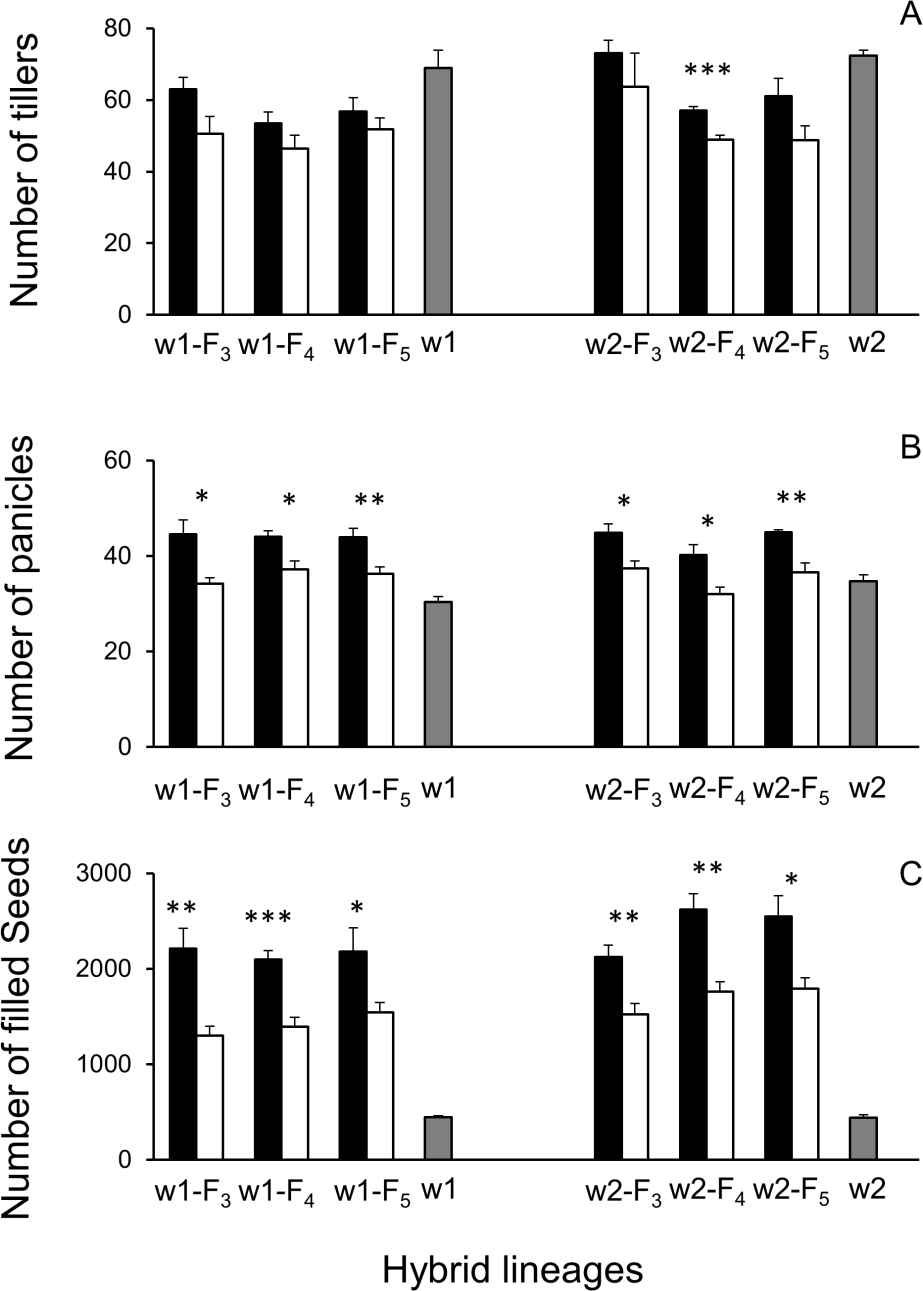
Fitness-related traits of F_3_–F_5_ crop (EP3)–wild rice (w1 or w2) hybrid lineages and their wild parents (gray columns) in pure-planting plots. **(A)** Average number of tillers per plant at the 120-day growth stage. **(B)** Average number of panicles per plant. **(C)** Average number of seeds per plant. Comparisons were made between GE (black columns) and non-GE (white columns) plants based on the independent-samples Student’s *t*-test. Bars represent standard error (n = 6 replicates). *p < 0.05, **p < 0.01; ***p < 0.001. GE, genetically engineered.

The independent-samples Student’s *t*-tests were performed to determine the differences in the fitness-related traits, including the number of tillers at the 120-day stage and the number of panicles and filled seeds per plant in the pure-planting plots. The obtained result showed a significantly greater (*p* < 0.05) number of panicles and filled seeds per plant in all the GE hybrid lineages than in their corresponding non-GE lineages (**Figure 1**). However, a significantly greater (*p* < 0.05) number of tillers per plant was only detected in the GE w2-F_4_ lineage than in its non-GE counterpart at the 120-day stage. No significant differences (*p* > 0.05) were detected between the other GE and non-GE hybrid lineages on this trait in the pure-planting plots (**Figure 1**).

**Figure 2:**
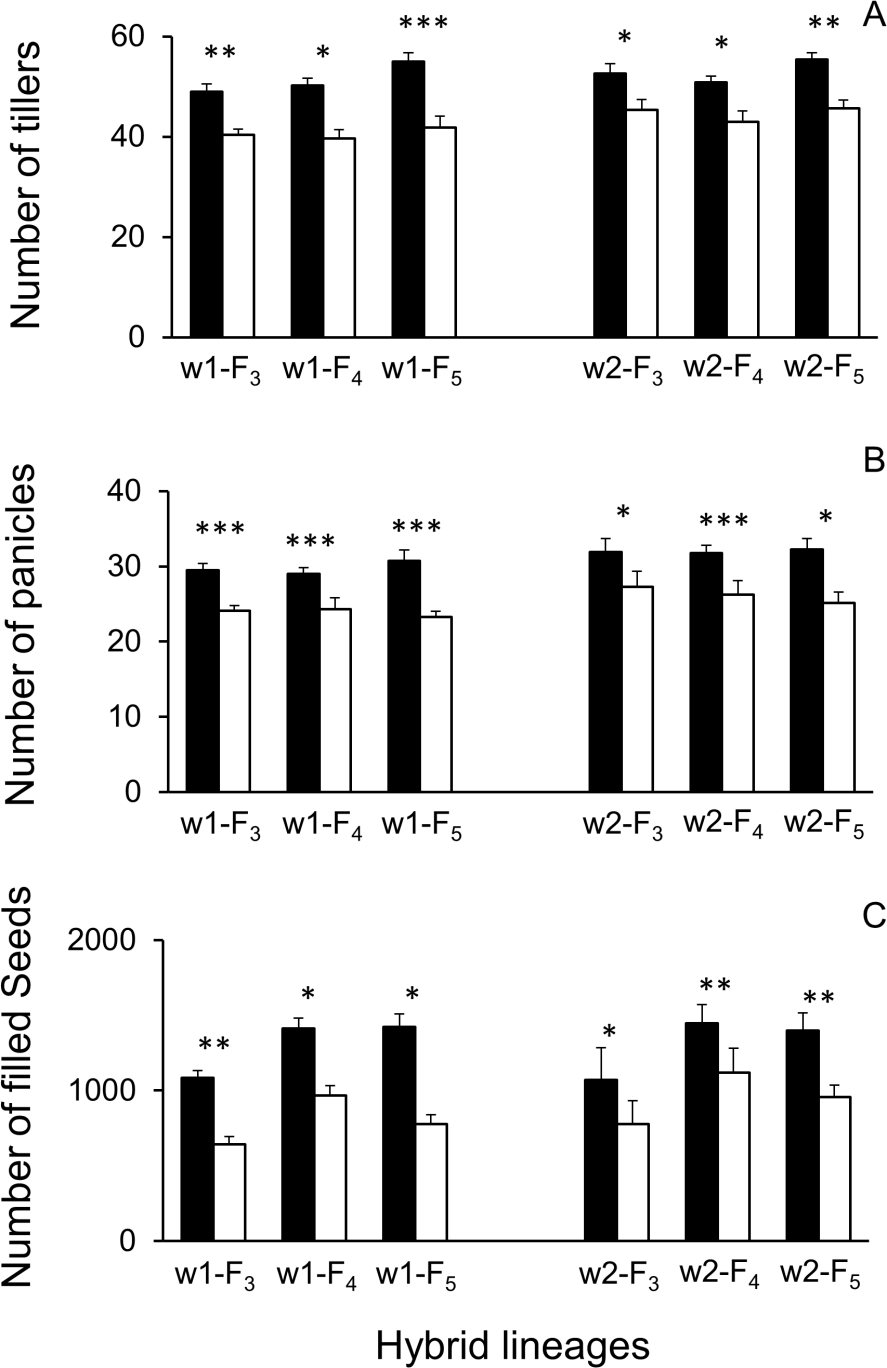
Fitness-related traits of F_3_–F_5_ crop (EP3)–wild rice (w1 or w2) hybrid lineages in mixed-planting plots. **(A)** Average number of tillers per plant at the 120-day growth stage. **(B)** Average number of panicles per plant. **(C)** Average number of filled seeds per plant. Comparisons were made between GE (black columns) and non-GE (white columns) plants based on the paired-samples Student’s *t*-test. Bars represent standard error (n = 6 replicates). *p < 0.05, **p < 0.01; ***p < 0.001. GE, genetically engineered.

The paired-samples Student’s *t*-tests were performed to determine the differences in the fitness-related traits in the mixed-planting plots. Results showed a significantly greater (*p* < 0.05) number of tillers at the 120-day stage, and the number of panicles and filled seeds per plant in all the GE hybrid lineages than in the non-GE lineages in the mixed-planting plots (**Figure 2**). Altogether, these results suggested that the transgene overexpressing *EPSPS* significantly increased the fitness benefits of the GE F_3_–F_5_ crop–wild rice hybrid lineages under the conditions of narrow spacing and relatively strong competition.

The independent-samples Student’s *t*-tests were also made to determine the differences in the mean performances of the fitness-related traits between the wild rice (w1 and w2) and their corresponding GE crop–wild rice hybrid lineages of different generations (F_3_, F_4_, and F_5_) in the pure-planting plots. For this analysis, the performance of the F_3_–F_5_ hybrids was averaged across generations, disregarding intergenerational differences, in order to assess whether the crop–wild rice hybrids possess any fitness advantage over their wild parental lines. All fitness-related traits measured in the common garden field experiment (**Table 1**) were included in the comparison. Results showed a significantly higher (*p* < 0.001) plant height at the 90- and 120-day stages, a greater number of panicles and filled seeds per plant, and higher 1000-seed weight. In addition, results also showed a significantly lower number (*p* < 0.01) of tillers per plant at the 90- and 120-day stages and greater over-winter plant survival ratios in the GE crop–wild rice hybrid lineages than in their wild rice parents (**Table 5**). The results suggested that the GE crop–wild rice hybrids had fitness advantages on the traits of plant height, the number of panicles and filled seeds per plant, and 1000-seed weight, although they had disadvantages on other traits, such as the number of tillers per plant and the over-winter plant survival ratios, compared with their parental wild rice plants.

**Table 5:** Comparison of the mean performances of the fitness-related traits between the wild rice populations and their GE crop–wild rice hybrid lineages of different generations (F_3_, F_4_, and F_5_) in the pure-planting plots based on the independent-samples Student’s *t*-test.

Traits	Mean performance of the wild rice and their hybrid lineages	
	Wild rice	Hybrid lineage^1^
w1 and their hybrid lineages		
Plant height at 60 days (cm)	57.6 ± 2.2^2^	64.0 ± 1.0 ^**3^
Plant height at 90 days (cm)	111.9 ± 7.8	147.2 ± 2.1 ^***^
Plant height at 120 days (cm)	132.0 ± 6.3	171.1 ± 2.1 ^***^
No. of tillers per plant at 60 days	8.5 ± 0.4	9.4 ± 0.6 ^NS^
No. of tillers per plant at 90 days	64.9 ± 5.1	44.9 ± 2.4 ^***^
No. of tillers per plant at 120 days	69.0 ± 5.0	57.8 ± 2.1 ^***^
No. of panicles per plant	30.3 ± 1.2	44.2 ± 1.2 ^***^
No. of filled seeds per plant	444.4 ± 18.3	2,164.5 ± 107.7 ^***^
1000-seed weight (g)	15.2 ± 0.3	21.3 ± 0.4 ^***^
Over-winter plant survival ratios (%)	93.2 ± 2.2	71.0 ± 2.6 ^***^
w2 and their hybrid lineages		
Plant height at 60 days (cm)	56.5 ± 2.2	59.1 ± 1.2 ^NS^
Plant height at 90 days (cm)	103.3 ± 3.2	143.1 ± 4.5 ^***^
Plant height at 120 days (cm)	127.5 ± 3.0	169.1 ± 3.0 ^***^
No. of tillers per plant at 60 days	7.1 ± 0.5	7.3 ± 0.4 ^NS^
No. of tillers per plant at 90 days	64.3 ± 1.4	49.1 ± 4.1 ^**^
No. of tillers per plant at 120 days	72.3 ± 1.6	63.9 ± 2.5 ^***^
No. of panicles per plant	34.7 ± 1.4	43.4 ± 1.1 ^***^
No. of filled seeds per plant	442.0 ± 29.1	2,432.7 ± 107.2 ^***^
1000-seed weight (g)	16.7 ± 0.3	20.3 ± 0.2 ^***^
Over-winter plant survival ratios (%)	94.3 ± 2.1	69.4 ± 2.8 ^***^

^1^Numbers followed by the averages indicate the standard error.

^2^Comparisons were made between the wild rice populations (n = 6 replicates) and their genetically engineered (GE) hybrid lineages of different generations (n = 18 replicates) in the pure-planting plots based on the independent-samples Student’s *t*-test.

^NS^ no significance; ^**^p < 0.01; ^***^p < 0.001.

### 3.3 Correlation between relative expression quantity of the *EPSPS* (trans)gene and fitness effect of GE crop–wild hybrid lineages

**Figure 3:**
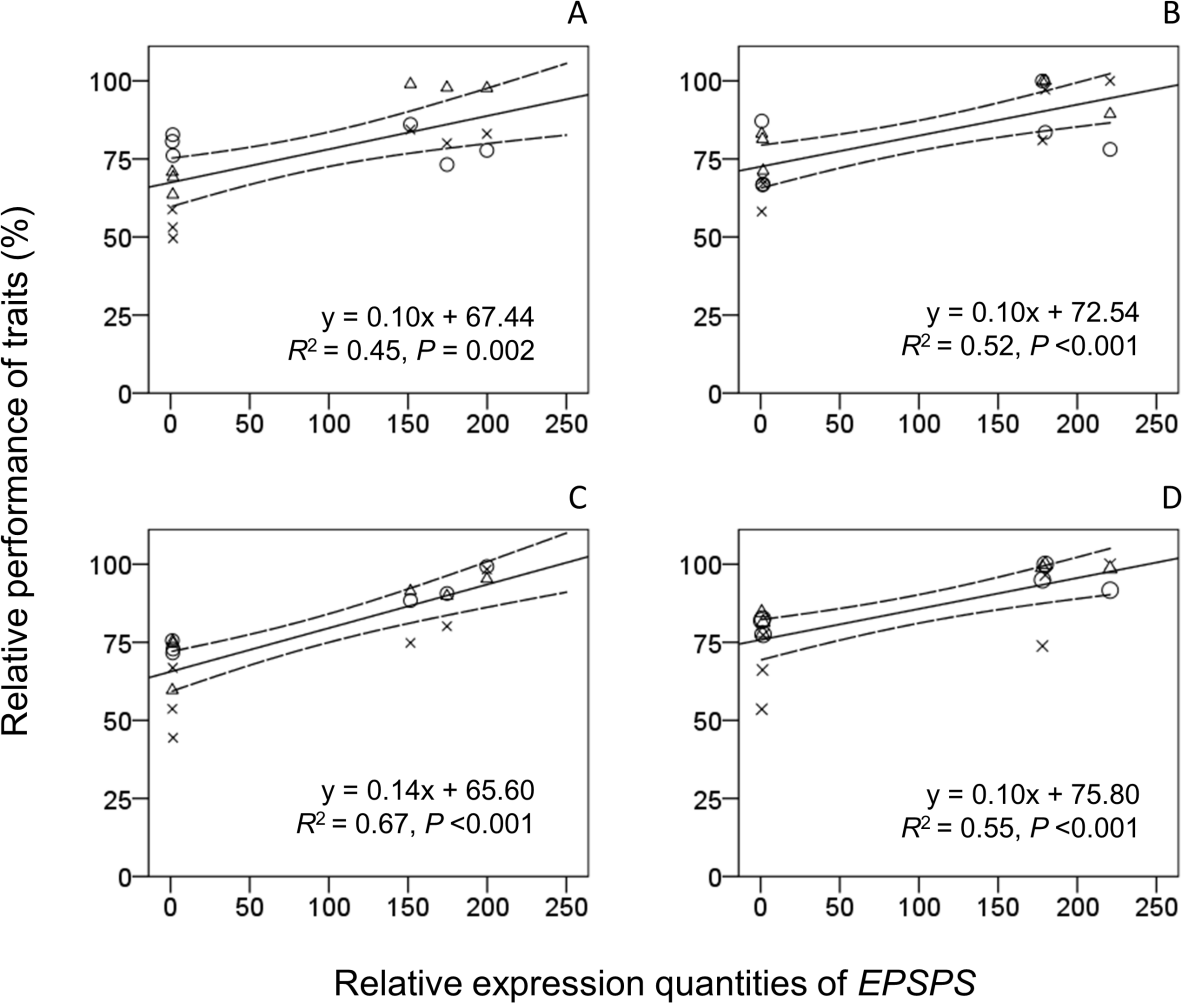
Correlation between the relative performance of fitness-related traits and the relative expression quantities (REQs) of *EPSPS* at the 90-day growth stage in crop (EP3)–wild rice (w1 or w2) hybrid plants (F_3_–F_5_). **(A, B)** EP3-w1 and EP3-w2 hybrid lineages in pure-planting plots. **(C, D)** EP3-w1 and EP3-w2 hybrid lineages in mix planting plots. Circles, relative fitness of tiller numbers per plant; triangles, relative fitness of panicle numbers per plant; crosses, relative fitness of filled seed per plant. Formulas indicate functions of regression lines. *R*^2^, coefficient of determination; *p*, level of significance in Pearson's test.

Pearson’s tests and linear regressions were performed to determine the correlation between the REQs of the *EPSPS* (trans)gene at the 90-day growth stage and the relative performance of the three key fitness-related traits with the stable fitness benefit caused by the *EPSPS* transgene. These fitness-related traits included the number of tillers at the 120-day growth stage and the number of panicles and filled seeds per plant under both the pure- and mixed-planting conditions. The obtained results showed significant positive correlations (*p* < 0.01, *R*^2^ = 0.45–0.67) between the REQs of the *EPSPS* (trans)gene and the relative performance of the three fitness-related traits in all the crop–wild rice hybrid lineages in both the pure- and mixed-planting plots (**Figure 3**). Altogether, these results suggested that the high level of *EPSPS* transgene expression played a significant role in enhancing the fitness benefits of the crop–wild rice hybrid lineages in different generations. In other words, the *EPSPS* transgene would cause considerable fitness benefits to the crop–wild rice hybrid lineages in advanced generations, as long as the *EPSPS* transgene is continuously overexpressing.

## 4 Discussions

### 4.1 Sustained high-level expression of the *EPSPS* transgene in the multi-generational GE crop–wild rice hybrid lineage

Our results obtained from this study indicated that the *EPSPS* transgene could significantly increase the REQs of EPSPS in plants of the advanced generations of GE crop–wild rice hybrid lineages (F_3_–F_5_). This finding was based on the continued finding of a high level of REQs from the overexpression of the *EPSPS* transgene in the advanced generations (F_3_–F_5_) of different hybrid lineages (EP3-w1 and EP3-w2) at the different plant growth stages (60 and 90 days). These results addressed our first question concerning the continued and consistent overexpression of the *EPSPS* transgene in crop–wild rice hybrid progenies of the advanced generations, which will determine the fate of the wild relative populations incorporated into this transgene (Lu and Snow, 2005; Lu, 2008; Lu and Yang, 2009). In other words, the incorporation of the transgene overexpressing the *EPSPS* into wild relative populations of GE rice varieties through crop-to-wild gene flow will permit the wild rice populations to gain the fitness benefit conveyed by the transgene with a considerably high level of *EPSPS* expression in a relatively long period.

Previous studies have already determined a considerably high frequency (>3%) of pollen-mediated gene flow from cultivated rice to its wild ancestor *O. rufipogon* (Song et al., 2003; Wang et al., 2006). However, the fate of the crop–wild hybrids and their progenies resulting from transgene flow depends on the successful persistence and spread of the transgene in the wild populations. If the transgene can be expressed normally and consistently bring any fitness benefit to the wild populations, it will promote the growth and development of the wild population, probably causing environmental impact. A study reported normal expression of a transgene in crop–wild rice hybrids and their F_2_ progenies (Xia et al., 2009). In addition, some studies have also reported the high expression level of *EPSPS* transgene in crop–weed rice F_2_ hybrid progeny and transgenic *Arabidopsis* plants containing the *EPSPS* transgene, both of which showed significant fitness benefit in the transgenic plants (Wang et al., 2014; Fang et al., 2018). Therefore, the assessment of long-term transgene expression and fitness benefit brought by a transgene(s) is critical for the determination of any environmental impact conferred by the transgene(s) (Lu and Snow, 2005; Lu, 2008; Lu and Yang, 2009). As such, we predict based on previous observations and our results from this study that consistent overexpression of *EPSPS* transgene in advanced generations of crop–wild rice hybrid progenies derived from a GE cultivated rice line will most likely increase the fitness benefit of the advanced hybrid populations steadily, causing the spread of the transgene in wild rice populations and consequently unwanted environmental impacts.

### 4.2 Enhanced fitness benefit of GE crop–wild hybrid lineages in advanced generations

Our results obtained from the common garden experiment in this study showed significantly increased values of fitness-related traits in different generations of the GE *EPSPS* crop–wild rice hybrid lineages, compared with those in the non-GE hybrid lineages. Generally, consistent fitness benefits brought by the *EPSPS* transgene were detected in the consecutive F_3_–F_5_ generations, suggesting that the *EPSPS* transgene remains effective in the advanced hybrid progenies. The above results addressed our second question regarding the stability of the transgene in advanced generations of crop–wild hybrid progenies with enhanced fitness benefits. To our knowledge, this is the first study documenting the fitness effect (benefit) caused by overexpressing the *EPSPS* transgene in the multigeneration crop–wild rice hybrid progenies.

The fitness benefit conferred by the transgene in crop–wild/weedy hybrid progenies is an important factor to determine whether or not the transgene will spread in wild populations through gene flow, resulting in ecological and environmental impacts (Burke and Rieseberg, 2003; Lu and Snow, 2005; Lu, 2008; Lu and Yang, 2009; Londo et al., 2010; Sagers et al., 2015). Some studies have reported the fitness-benefit effect of *EPSPS* transgene in cultivated rice (Achary et al., 2020) in F_1_ or F_2_ hybrids of crop–wild/weedy rice hybrids (Wang et al., 2014; Yang et al., 2017b) and in GE *Arabidopsis* plants (Fang et al., 2018). These studies have generally supported the observations that overexpressing the *EPSPS* transgene would significantly increase the fitness of the plants. However, no study has addressed the question of whether the fitness benefit caused by overexpressing the *EPSPS* transgene can be consistently passed to the advanced generations of hybrid progenies, which is also important to determine the long-term ecological and environmental impact from transgene flow (Lu et al., 2016; Yang et al., 2015). Our results obtained from this study clearly demonstrate that the fitness benefit conveyed by the *EPSPS* transgene will be passed to the advanced generations of crop–wild rice hybrid progenies or populations containing this transgene. Although ANOVA results indicated variation in fitness traits—including plant height, tiller number per plant, filled seeds per plant, 1000-seed weight, and over-winter survival rate—no significant interaction between the transgene and generation (T × G) was detected for most traits. This suggests that the fitness effect of the transgene remained consistent across generations. Moreover, we observed no clear pattern of fitness variation across the F_3_–F_5_ generations. Therefore, we propose that the transgene effect persists in advanced generations, despite some variability in fitness during propagation.

Furthermore, while our study focused on the effect of the EPSPS transgene on vegetative growth and seed production, previous research has reported significant advantages in seed germination and longevity for crop–wild rice and soybean hybrid seeds carrying the EPSPS transgene (Yang et al., 2017b; Yook et al., 2021; Zhang et al., 2023). These findings suggest that the fitness benefits conferred by the EPSPS transgene extend across the entire life cycle of hybrid plants.

Noticeably, the comparison between the GE hybrid progenies and their parental wild rice populations indicated that the GE crop–wild rice hybrid progenies had a considerable advantage in traits related to their vegetative growth and sexual reproduction, including the plant height at 90 and 120 days, the number of panicles and filled seeds per plant, and 1000-seed weight. However, disadvantages were also detected on the other traits, such as the number of tillers per plant and the over-winter plant survival ratios, in the GE hybrid progenies, compared to their parental wild rice populations. In fact, the GE hybrid progenies will be under competition not only with the non-GE hybrid counterparts but also with their wild parental populations under different environmental conditions. For example, compared with the wild parental populations (*O. rufipogon*), the GE hybrid progenies containing the *EPSPS* transgene still showed considerable disadvantages in regeneration ability estimated using the over-winter plant survival ratios. In contrast, another study reported the significantly increased longevity and survival of seeds from F_4_–F_6_ GE crop–wild rice hybrid lineages in the soil seed banks containing the same *EPSPS* gene (Jiang et al., 2021). Therefore, a comprehensive fitness assessment procedure, including the GE, non-GE hybrid progenies, and wild parental populations, should be taken into consideration in determining the impact of a transgene in wild or weedy species populations for long-term environmental biosafety.

### 4.3 Consistent *EPSPS* overexpression increased fitness of GE crop–wild hybrid progenies

Results based on the correlation analyses showed a significant positive correlation between the *EPSPS* REQs and key fitness-related traits, including the number of tillers, panicles, and filled seeds per plant. These results suggest that consistent *EPSPS* overexpression enhanced the fitness benefit in different generations of GE crop–wild hybrid progenies and also addressed our third question in the Introduction section concerning whether the overexpression of the *EPSPS* (trans)gene is correlated with the enhanced fitness benefit in the GE hybrid progenies.

EPSPS (EC 2.5.1.19) acts as the key enzyme in plant for biosynthesizing the aromatic amino acids (i.e., tryptophan, phenylalanine, and tyrosine) in the shikimate pathway, which plays an essential role in plant growth and development by producing plant hormone (auxin), lignin, chlorophyll, and other secondary metabolites (Tzin et al., 2009; Maeda and Dudareva, 2012). The critical role of downstream products from the shikimate pathway suggests the importance of overexpressing the *EPSPS* gene that enhances the fitness of plants by promoting the biosynthesis of the above amino acids and other secondary metabolites. Studies of hybrid progenies derived from crosses between GE cultivated rice and wild or weedy rice populations have indicated that the overexpression of the *EPSPS* transgene significantly increased the content of tryptophan, lignin, and chlorophyll (Wang et al., 2014; Achary et al., 2020; Ma et al., 2020; Wu et al., 2020) and consequently brought fitness benefits to the hybrid progenies containing the transgene (Wang et al., 2014; Yang et al., 2017a; Achary et al., 2020). Another study also reported that GE *Arabidopsis* plants overexpressing the *EPSPS* transgene produced more auxin (indole-3-acetic acid) and seeds (Fang et al., 2018). As a consequence of the enhanced metabolism conferred by the *EPSPS* transgene, a significant increase in carbon consumption was observed in *Arabidopsis* and rice, resulting in higher total plant biomass (Sun et al., 2023). Therefore, we conclude based on these results that the fitness benefit brought by the *EPSPS* transgene in multigeneration (F_3_–F_5_) crop–wild rice hybrid progenies observed in this study, including the increased tiller and seed number per plant, is most likely due to the high expression level of the *EPSPS* transgene, which significantly affected the normal processes of plant metabolism and carbon fixation. In addition, consistent overexpression of the *EPSPS* transgene in our study resulted in the continued fitness benefit in the F_3_–F_5_ generations of crop-wild hybrid progenies. Therefore, we expect that the long-term and sustained fitness benefit in more generations of crop-wild/weedy hybrid populations causes environmental impacts.

### 4.4 Implications in assessing long-term environmental impacts caused by the transgene overexpressing *EPSPS*

The results of this study demonstrated the consistent fitness benefit in GE crop–wild rice hybrid progenies caused by sustained overexpression of the *EPSPS* transgene. This finding indicates that the advanced generations of GE crop–wild or crop-weed rice hybrid progenies containing the overexpressing *EPSPS* transgene will consistently have a fitness advantage, resulting in the persistence and spread of the transgene in the wild or weedy populations because of its competitive advantages. During this process, the *EPSPS* transgene together with the crop alleles linked to the transgene may gradually replace the endemic alleles in wild and weedy relative populations via the genetic swamping or sweeping effect (Lu and Yang, 2009; Todesco et al., 2016; Wang et al., 2017), leading to the loss of the genetic diversity in wild or weedy gene pools (Lu and Snow, 2005; Lu and Yang, 2009; Cai et al., 2023). In addition, many studies have indicated that some weedy rice populations were evolved from the natural hybridization or gene flow between cultivated and wild rice (Olsen et al., 2007; Yao et al., 2020; Cai et al., 2023). The published reports, together with results from this study, strongly suggest the potential environmental impact from crop-to-wild rice and crop-to-weedy rice transgene flow if this transgene, particularly the herbicide tolerance transgene, can consistently and significantly increase the fitness benefit of the gene flow, resulting in hybrid progenies. Therefore, the long-term risk assessment and monitoring of the crop–wild or crop–weed rice hybrid populations containing such a transgene consistently overproducing EPSPS should be performed for the environmental biosafety impact.

In addition, based on our findings of the positive correlation between the REQs of the *EPSPS* (trans)gene and the values of fitness-related traits of crop–wild rice hybrid plants in different generations, here, we also propose a more practical method of the long-term environmental biosafety assessment, particularly for the GE crops containing the *EPSPS* overexpressing transgene. In this method, the REQs of the *EPSPS* (trans)gene are accurately determined, and the REQ values can be applied to estimate the potential fitness of crop–wild hybrid plants in different generations. By doing so, we can avoid conducting extensive common garden field experiment to collect the fitness data and save time and resources for environmental biosafety assessment. Whether the assessment method is functioning and practical still needs to be examined and validated by more experiments.

In conclusion, results obtained from this study demonstrated the significantly higher expression level of the *EPSPS* (trans)gene in the advanced generations (F_3_–F_5_) of crop–wild rice hybrid progenies overexpressing the *EPSPS* transgene. Consequently, the consistent and higher REQs of the *EPSPS* transgene resulted in increased fitness benefit continuously in F_3_–F_5_ crop–wild rice hybrid progenies under the herbicide-free conditions. We therefore predict that the consistent fitness benefit conferred by the *EPSPS* transgene will be present in even more advanced generations of crop–wild rice hybrid progenies. Such transgene flow may result in the persistence and spread of the *EPSPS* transgene in wild rice populations, causing the loss of genetic diversity in the wild rice populations and unwanted long-term environmental impact. Based on these findings, we think that it is necessary to conduct more studies on the fitness effect conferred by the *EPSPS* transgene for the long-term environmental biosafety impact caused by gene flow from crops to their wild relatives. We also propose a novel method of the environmental biosafety assessment for GE crops containing the *EPSPS* overexpressing transgene by accurately determining REQs of the *EPSPS* (trans)gene to estimate the potential fitness of crop–wild hybrid plants conferred by the transgene in different generations.

## Data Availability

The original contributions presented in the study are included in the article/**Supplementary Material**. Further inquiries can be directed to the corresponding author.

## Supplementary material

Supplementary Figure 1 Schematic illustration showing the possible trends of fitness effect conferred by the transgene in crop−wild/weed hybrids. Solid line, transgenic hybrids; dotted line, non-transgenic hybrids. **(A)** fitness of the transgenic hybrids remains stable in the sequent generations, resulting consistent long-term ecological and environmental impacts; **(B)** fitness of the transgenic hybrids decreases in the sequent generations, resulting temporary impacts.

Supplementary Figure 2 Production of crop−wild (*Oryza sativa* L. and *Oryza rufipogon* Griff.) rice F_3_-F_5_ hybrid lineages. ++, +−, and −− indicate transgenic (GE) homozygous, transgenic heterozygous, and non-transgenic (non-GE) homozygous, respectively. F_3_-F_5_ hybrid progeny were used to examine the EPSPS expression and fitness related traits in the common garden experiment.
