# Supplementary figures and images for: Continued overexpression of EPSPS transgene enhances fitness in multigeneration crop–wild rice hybrids and its long-term environmental impact
Source: Front Plant Sci. 2026 Mar 9;17:1707859. doi: 10.3389/fpls.2026.1707859 (PMC13006699; doi:10.3389/fpls.2026.1707859)

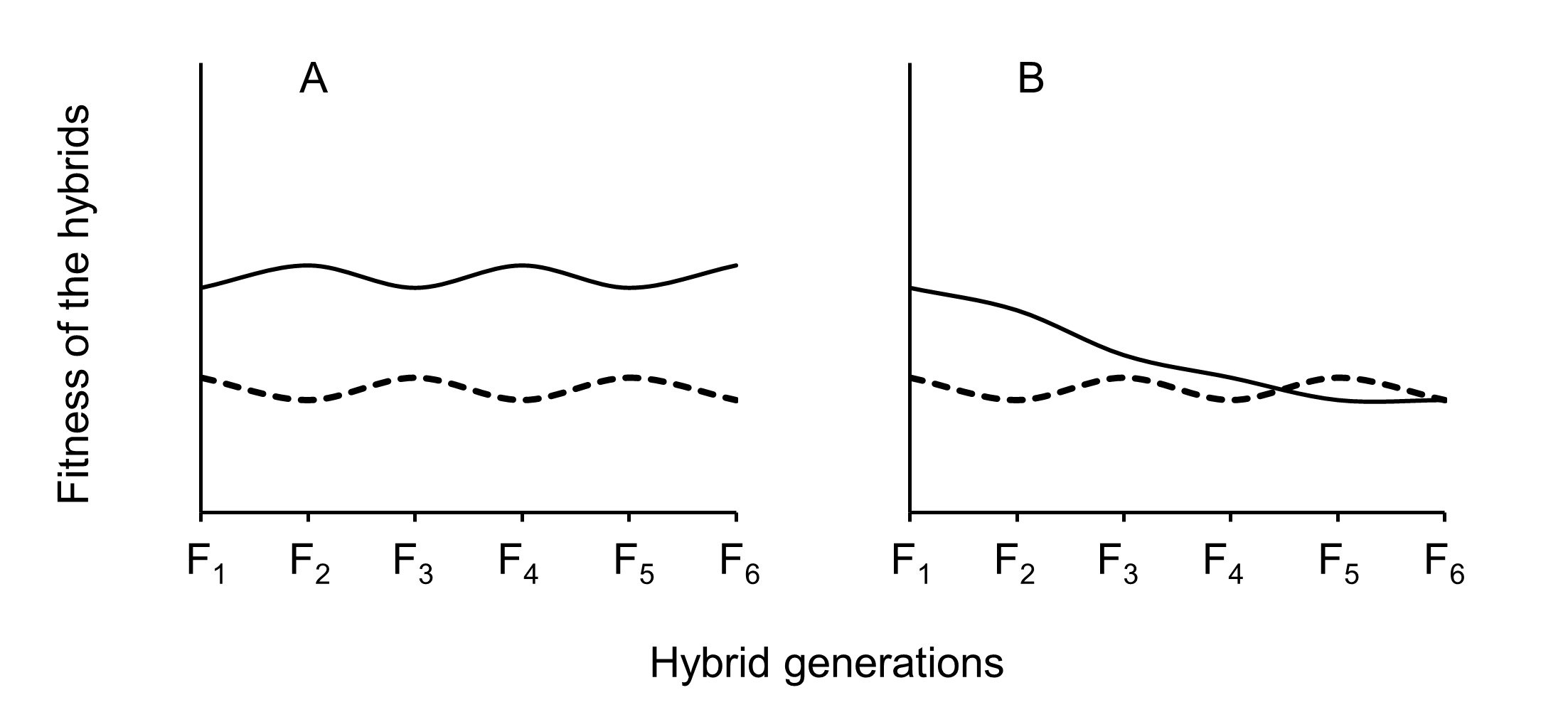

Supplement: Supplementary Figure 1 — Schematic illustration showing the possible trends of fitness effect conferred by the transgene in crop−wild/weed hybrids. Solid line, transgenic hybrids; dotted line, non-transgenic hybrids. (A) fitness of the transgenic hybrids remains stable in the sequent generations, resulting consistent long-term ecological and environmental impacts; (B) fitness of the transgenic hybrids decreases in the sequent generations, resulting temporary impacts. [file Image1.tif]

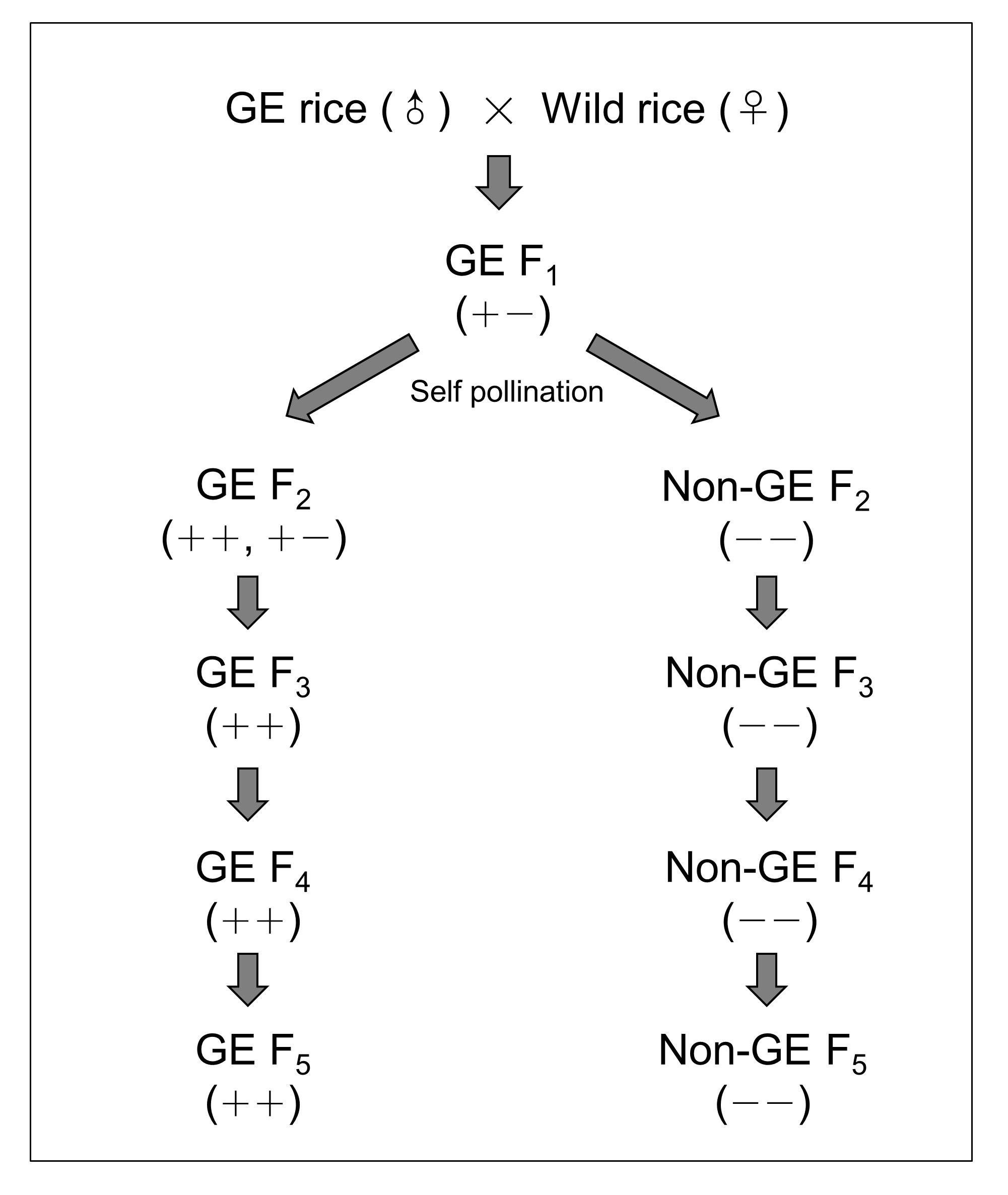

Supplement: Supplementary Figure 2 — Production of crop−wild (Oryza sativa L. and Oryza rufipogon Griff.) rice F3-F5 hybrid lineages. ++, +−, and −− indicate transgenic (GE) homozygous, transgenic heterozygous, and non-transgenic (non-GE) homozygous, respectively. F3-F5 hybrid progeny were used to examine the EPSPS expression and fitness related traits in the common garden experiment. [file Image2.tif]
